# Intelligent Alzheimer's diagnosis and disability assessment: robust medical imaging analysis using ensemble learning with ResNet-50 and EfficientNet-B3

**DOI:** 10.3389/fmed.2025.1619228

**Published:** 2025-09-02

**Authors:** Arpanpreet Kaur, Fehaid Salem Alshammari, Ateeq Ur Rehman, Salil Bharany

**Affiliations:** ^1^Chitkara University Institute of Engineering and Technology, Chitkara University, Rajpura, Punjab, India; ^2^Department of Mathematics and Statistics, College of Science, Imam Mohammad Ibn Saud Islamic University (IMSIU), Riyadh, Saudi Arabia; ^3^King Salman Center for Disability Research, Riyadh, Saudi Arabia; ^4^Computer Science and Engineering, Saveetha School of Engineering, Saveetha Institute of Medical and Technical Sciences, Chennai, Tamil Nadu, India; ^5^Applied Science Research Center, Applied Science Private University, Amman, Jordan

**Keywords:** Alzheimer's disease, neurodegenerative disorder, deep learning, MRI analysis, ResNet-50, EfficientNet-B3, ensemble model, feature extraction

## Abstract

Neurodegenerative disorder Alzheimer's disease (AD) has progressive characteristics and leads to severe cognitive impairment that reduces life quality. Disease management along with effective intervention depends on the detailed diagnosis conducted early. The proposed framework builds an ensemble system from ResNet-50 and EfficientNet-B3 to conduct automated AD diagnostics by processing high-resolution Magnetic Resonance Imaging (MRI) images. The proposed model uses ResNet-50 to extract features coupled with EfficientNet-B3 as its robust classifier which achieves high accuracy alongside generalization performance. A large, high-quality dataset comprising 33,984 MRI images was used, ensuring diverse representation of different disease stages: the study included participants with four dementia stages organized as Mild, Moderate, Non-demented, and Very Mild Demented. The research applied several comprehensive data preprocessing methods combining normalization steps with rescaling algorithms alongside noise elimination techniques to achieve enhanced performance. Performance tests on the model required examination of accuracy along with precision and recall metrics and F1-score and ROC curve area measurements. The ensemble model delivered remarkable overall accuracy reaching 99.32% while surpassing separate deep learning architectures. The confusion matrix evaluation results showed superb classification results for Mild and Moderate stages along with non-dementia cases while maintaining minimal Wrong choices in Very Mild Demented cases. Experimental findings demonstrate the strength of deep learning algorithms to detect AD disease stages accurately. The robust and accurate performance of the proposed model indicates it has potential for use in medical environments to support radiologists in their work of early-stage AD screening and treatment development. Additional research in diverse clinical environments will strive to optimize and validate the model so it can meet real-world diagnostic requirements for medical use.

## 1 Introduction

Alzheimer's disease (AD) is a primary neurodegenerative disease that is responsible for 60%−70% of all dementia cases across the globe, it results in progressive impairment of cognitive and memory function, and overall physical disability mainly in old age. The disease is defined clinically by the deposit of amyloid plaques and neurofibrillary tangles in the brains, leading to the gradual decline in brain volume, and resulting in confusion, poor judgement, language disorder, personality changes, and the inability to carry out activities of daily living (1). To date, aging is still the biggest risk factor for developing AD, but there are also genetic factors, unhealthy life styles, cardiovascular diseases and physical environments that affect the development as well as the progress of AD (2). To date, there is no known cure for Alzheimer's disease but major advancements in medical research have provided methods of managing the disease, these include; cholinesterase inhibitors, memantine, health and safety promotion through changes in diets and coming up with strict exercise regimes that can reduce deterioration of the patient's condition (3). Prior to the publication of DSM IV-Tre quantitative diagnosis of Alzheimer's disease primarily depended on clinical assessment, patient history, and neuropsychological assessment that even though still today have their utility, were reported to provide low sensitivity in early diagnosis of Alzheimer's disease as well as being time consuming and labor intensive. Also, Magnetic Resonance Imaging (MRI) and PET scans have been used to detect abnormalities in the brains of mentally ill patients, although these approaches lack high accuracy when no computational tools are applied (4). Over the last few years, the incorporation of deep learning methods in medical imaging has definitely advanced diagnosis, particularly for Alzheimer's disease as a more precise, fast, and less error-prone approach (5).

Among these, Convolutional Neural Networks (CNNs) have shown exceptional performance in efforts to diagnose MRI patterns that point toward AD, all while surpassing conventional machine learning models by learning features from raw image data. In the context of Alzheimer's disease, the required diagnostic tools are significantly more diverse and refined; this is why ensemble deep learning models have recently become popular as they unite the results of several architectures in one model (6). As for the CNN model selection, two advanced structures including ResNet-50 and EfficientNet-B3 have become the most popular pro forma architectures in recent years due to the higher image classification performance. The vanishing gradient problem is solved through using the ResNet architecture of a deep residual network of 50 layers; deeper networks converge well while capturing details of the images at the same time (7, 8). On the other hand, EfficientNet-B3 uses compound scaling method to control the network depth, width, and so on, making it highly efficient and accurate to extract features with little computational need. Thus, the ensemble of ResNet-50 and EfficientNet-B3 models, where the weaknesses of each of them are masked, and the strengths are combined, contributes to increasing the efficiency of diagnostics compared to using only such architectures and increases the robustness when detecting subtle abnormalities in MRI scans. The main goal of this research is to enhance a deep learning model for distinguishing between the Alzheimer's disease and the Normal Cognitive status by integrating ResNet-50 and EfficientNet-B3 models for MRI data. This approach operates in an attempt to overcome the recognized deficiencies of conventional diagnostic check techniques for AD through the development of an efficient diagnosis system that would be automated, accurate, and fairly easy to implement in the different human populations at the various stages of the disease development (9). In addition, the problem statement focuses on the requirement of an accurate diagnostic tool to differentiate between distinct phases of Alzheimer's disease with robust performance, despite data imbalance, MRI scan noise, and variation (10). Therefore, the major contributions of this study are the development of an ensemble model that comprises ResNet-50 and EfficientNet-B3, an assessment of the performance of the proposed ensemble model against existing deep learning architectures, and a proof of the usefulness of the suggested model in enhancing the diagnostic accuracy of Alzheimer's disease classification. Several works have been extensively conducted on AD detection using standalone CNNs, CNNs with Attention Mechanisms, Ensemble of CNNs and the hybrid of them; their performance is sometimes constrained by a limited number of available diagnostic samples, non-normative database information, and high computational costs (4, 8). For example, Ajagbe et al. (3) and Shirbandi et al. (6) pointed out that applying CNN-based models in MRI-based classification is promising; however, that architectures should be improved to learn deeper and abstract features. Finally, the studies by Sorour et al. (8) and Mujahid et al. (7) showed that the setup based on the ensemble learning is extremely valuable for the detection of AD, as the results of multiple models enhanced positive prediction and diminished the numbers of false-positives. Thus, basing on these achievements, the development of our proposed model is intended to fill the gap in the identified scientific studies and integrate the advantages of ResNet-50 and EfficientNet-B3, including their residual learning ability and computational efficiency. Furthermore, the given work uses techniques like data augmentation and employs adaptive learning to deal with issues that are hard to solve for, including overfitting and imbalance, in order to have a high model accuracy on various MRI datasets (7). The reason as to which ResNet-50 and EfficientNet-B3 were selected for the experiment is because these two architectures have demonstrated good performance across multiple tasks and are robust combinations of feature extraction and classification (8). Based on its deep residual connections which allow the model to learn complex features, ResNet-50 is well-suited to this task, whereas EfficientNet-B3 which incorporates optimized scaling for efficient computations is equally efficient and accurate for the task at hand. This combination is specifically advantageous for medical imaging applications where the minor differences have to be identified between the structures of normal brains and that of the AD patients (6). Moreover, ensemble learning is beneficial in increasing the generalizability of the model, since the combination of more predictions means decreasing the model bias and variance and thus, increasing the diagnostic reliability (7). Finally, this paper intends to make a positive contribution to the available body of knowledge on Alzheimer's disease by proposing a new, yet highly effective, deep learning structure that encompasses the best facets of the ensemble learning technique to deliver the highest possible diagnostic accuracy. Of critical value and practical applicability, the proposed model can help clinicians make quick and precise diagnosis decisions, which will lead to earlier diagnosis, target treatment plans, and enhanced patient care (2, 8).

In this context, this study contributes to fill the gap of the current diagnostic techniques in Alzheimer's disease and to establish the base for future studies that will promote the creation of new, available and reliable tools with deep learning for Alzheimer's disease diagnosis in magnetic resonance images. This work couples two important elements for the construction of an effective diagnostic test for Alzheimer's disease based on high classification accuracy and explainability. Section 2 gives an extensive literature review of the existing diagnostic conventional approaches, deep learning in neuroimaging. Next, in Section 3, the method is described, more specifically, details about the dataset, the preprocessing of MRI scans, the architecture of the proposed ensemble model based on ResNet-50 and Efficient Net B3. In Section 4, the authors report the findings analyzing the effectiveness of the ensemble model and taking them up against the other classification models. Section 5 contains a discussion of the study's results and their potential, possible clinical uses of the proposed model, its weaknesses, and potential improvements for future work. Also in Section 6, the conclusion of the paper points to the contributions of the study and the implication of applying the proposed approach to timely diagnosis of Alzheimer's disease.

## 2 Literature review

Alzheimer's disease (AD) classification has received a considerable amount of focus in the medical research sector mainly due to the development of new approaches such as deep learning, which have indicated that they can outperform conventional diagnostic approaches. The two best performing deep learners in this study are the Convolutional Neural Networks (CNNs), specifically ResNet-50 and EfficientNet-B3 reveal promising features for efficient AD diagnosis from brain MRI scans. The subjects of Raza et al.'s (11) study involved segmentation and classification of MRI images of Alzheimer's disease employing transfer learning (TL) and proposed particular CNNs. The approach works on images that segment objects as divided by the brain's Gray Matter. Rather than training from the ground up, there existed a pre-trained deep learning model, to which the process proceeded as transfer learning. The model was compared at 10, 25, and 50 epochs and the mean accuracy was found to be 97.84%. Ironically, transfer learning and segmentation techniques stand as prominent methodologies in a comprehensive framework of medical imaging analysis in diagnosing Alzheimer's disease this study shows the enhancement of accuracy (11). Sharma et al. presents a machine learning model based on transfer learning (TL) and permutation-based voting classifiers for Alzheimer's detection from MRI images. DenseNet-121 and DenseNet-201 extract features in phase one and phase two has classifiers such as support vector machine, Naïve Bayes and XGBoost to classify. Therefore, in the voting mechanism the final predictions are improved with accuracy of 91.75%, specificity of 96.5% and F1-score of 90.25. The model was trained from scratch using a Kaggle data set consisting of 6,200 images in four dementia classes. Mentioned results are completely compatible with the statements and show the higher effectiveness of the offered model compared with state-of-the-art methods; thus, there is perspective to consider the proposed model for usage in clinician applications for Alzheimer's disease identification (12). The authors Zhang, Zhang, Du, and Wang (13) in their study proposed an enhanced neural network known as ADNet from the VGG-16 model for detection of Alzheimer's diseases applying 2D MRI slices. Those modifications consist of depthwise separable convolution to decrease the number of parameters; however, the model uses ELU activation to avoid the problem of exploding gradients; the model also incorporated an SE module for effective feature recalibration. Similarly, training is combined with auxiliary tasks: regression of clinical dementia and mental state score. Experimental results proved that the proposed approach gives 4.18% higher accuracy of AD compared with cognitively normal (CN) and 6% of MCI accuracy compared with CN than the VGG16 model. These outcomes indicate that multitask learning solutions and better architecture for the neural network may help ADNet to support early Alzheimer's detection. Solano et al. (14) uses a three dimensional DenseNet model for the detection of Alzheimer's disease using Magnetic Resonance Imaging (MRI). Using the proposed deep neural network classifier, an overall accuracy of 0.86, sensitivity of 0.86, specificity of 0.85, and the area under the ROC curve (micro-average) of 0.91 for five disease stages. Focusing on the ability to produce replicable results, the approach uses only the tools available freely online, which means it should be more easily implemented in poorer countries as well. This approach helps to show that deep learning is useful in medical diagnosis and the equitable distribution of technology for installation and use. Carcagnì et al. (15) investigate the performance of CNNs and the adaptive self-attention mechanism for identifying Alzheimer's using brain MRI data. In particular, the study utilizes deep learning methods in improving the detection accuracy and speed of Alzheimer's disease, through exploiting the features of CNN, through a feature extraction step and exploiting self-attention to learn the long-range dependencies. In addition, proofs reveal a vast scope for the use of some automated diagnostic tools to have a high sensitivity and specificity compared with conventional practices. The work focuses on the implementation of the new AI models in the early diagnosis and effective individualized approach to the disease, providing a solid base for non-invasive and horizontally scalable dementia diagnostics (16). In recent years, deep learning proved to be a valuable approach in analyzing genomes, responding to the large and dependent features' patterns and correlations. The recent innovations include variation in model structures, paradigms of model establishment, and techniques of model decoding all focused on the prophetic models of genetic variants and their influence on the disease causation. In such context, this review addresses how genomic deep learning techniques remain rather flexible for disease-oriented investigations with reference to neurodegenerative disorders including Alzheimer. It uses primarily the articles on Alzheimer's disease and considers more general methods, explaining the potential value of these approaches. To the best of our knowledge, the review conducted by Jo et al. aimed at reviewing future research directions at the crossroads of neurodegeneration, genomics, and deep learning (16). Deep learning has emerged as an essential element of genomic analysis because of its capability to handle large genomic data by identifying the diverse relationships between them. Progress includes the following new trends in models: model architecture, model development philosophies, and model interpretation techniques for estimating the effects of genetic variants on disease progression. This review shows how to incorporate genomic deep learning methods into disease-specific models with an emphasis on neurodegenerative diseases such as Alzheimer's. It focuses on Alzheimer's literature and where it identifies more general methodological approaches, it explores their suitability. In addition, Qui et al. have discussed directions for future work involving neurodegeneration genomics, and deep learning (17). Hazarika et al. compares different deep learning (DL) models in AD classification using brain Magnetic Resonance (MR) images collected from the Alzheimer's Disease Neuroimaging Initiative (ADNI) dataset. However, the DenseNet-121 model showed the highest accuracy of 88.78%, a bit slower than the others because of the extensive convolutions. Thus, to overcome this kind of limitation, the authors suggested a new DenseNet-121 structure, where instead of the conventional convolutional layers, the depth-wise convolutional layers should be used. These optimizations improved computational and accuracy rates making the average accuracy to be 90.22%. The results discussed above imply future possibilities of depth-wise convolution in enhancing the DL-based AD classification models (18). In their paper, Helaly et al. describes a system for early detection of Alzheimer's disease (AD) and multi-stage classification with the help of convolutional neural networks (CNNs). Two methods are explored: specifically, the use of 2D and 3D CNNs for structural images, and apply transfer learning with VGG19 to improve the classification performance. Therefore, based on the ADNI dataset, the highest precision rate established was 93.61% in 2D; 95.17% in 3D, and 97% in VGG19. A web application helps in diagnosing and staging AD remotely, and improving health care access during COVID-19. The approach is simple and less computationally demanding, and the method's performance is stable and suitable for medical applications based on its evaluation on nine criteria (19). Jo et al. employed the 3D convolutional neural networks (CNN) and layer-wise relevance propagation designed to diagnose AD using tau PET scans. MCI using the proposed model he has come up with a result of 90.8% accuracy by using AD and cognitively normal (CN) subjects. Using information from voxel-wise analysis the key regions identified were hippocampus, thalamus, and entorhinal cortex. Probability of AD, calculated from cognitive measures, was associated with medial temporal tau deposition in MCI, proving useful in detection at this stage (20). Table 1 below shows the state of art comparison.

**Table 1:** Comparison with state of art.

**Reference**	**Technique used**	**Advantages**	**Disadvantages**
Sharma et al. (2022) (12)	Hybrid artificial system (HTLML) for Alzheimer's disease diagnosis	Finally, the use of multiple Artificial Intelligence techniques for a better result	They proposed that complexity manifested in hybrid models could result in longer time taken during training and high computational costs
Qiu et al. (2020) (17)	A clear and understandable deep learning structure	Used for explaining model adult human decision making	
Jo et al. (2020) (20)	Using residual deep learning on tau PET imaging	Concentrates in the identification of tau protein images in Alzheimer's	May be tuned to small fluctuations in MRI data
Solano-Rojas and Villalón-Fonseca (2021) (14)	A DenseNet neural network for early identification of Alzheimer's disease	A less expensive method with reasonable efficiency for early detection	Lacks capability of real time and high processing speed for 3D data
Jo et al. (2022) (16)	Application of deep learning for the analysis of genetic variants	It allows the analysis of massive genetic data to classify Alzheimer's	Is highly dependent on the availability of large high quality genotype data for use in training
Hazarika et al. (2022) (21)	Different Deep Learning Architectures for Alzheimer's Classification	Compared and contrasted several models, toward the decision-making process of selecting the right approach	Some of these techniques may compromise the model's accuracy or, sometimes, make it less complex
Helaly et al. (2022) (18)	AI based early diagnosis of Alzheimer's disease	Another stamina is early identification abilities since the program detects omissions at the beginning	Mixed evidence provided by models; models need to be chosen more carefully
Raza et al. (2023) (11)	Preprocessing and feature selection in Alzheimer's disease identification	Utilizes pre-trained models that mostly help to decrease the time and amount of training data needed	Some of native to the domain features might not be recognized by the pre-trained models
Carcagnì et al. (2023) (15)	CNN and self-attention learners	Proper to extract features from the brain MRI images	Self-attention mechanism may be costly
Zhang et al. (2024) (13)	This proposal addresses multi-task learning with an enhanced or modified version of a neural network	Multi-talented and able to work on a number of projects at once, hence increasing productivity	Complexity in models often leads to over fitting and these models will need large data sets

## 3 Proposed methodology

**Figure 1:**
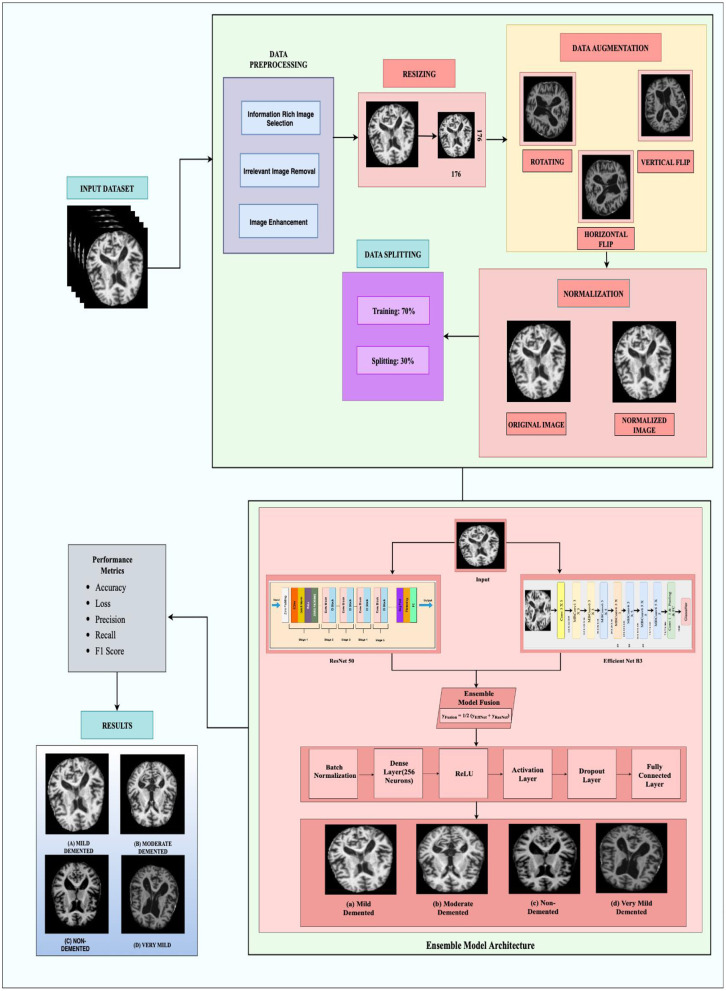
The framework of proposed methodology.

On the same note the proposed methodology outlines a comprehensive framework of Alzheimer's disease diagnosis. First, a clear overview of the dataset is provided, including its characteristics, which is diverse, clean and has high quality ground truth labels to enable accurate training and testing. Normalization, rescaling, center cropping, and elimination of noisy regions also prepares the data to be in the right standard. Each of these transformations enhances model robustness and, at the same time, can help increase its ability to generalize. The diagnostic framework involves an ensemble model of ResNet-50 and EfficientNet-B3 networks which are the best for the feature extraction and the classification, respectively. Moreover, evaluation criteria by accuracy, precision, recall, F1-score, and area under the ROC curve are used to provide more detailed analysis of the performance of the model. A general idea of the proposed methodology flowchart is presented in Figure 1 below.

### 3.1 Dataset description

**Figure 2:**
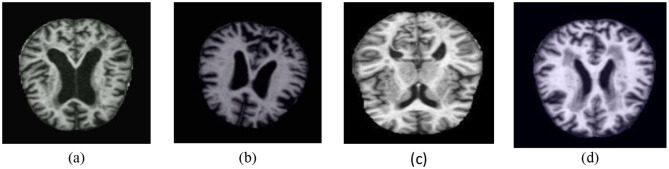
Dataset classes: **(a)** Mild Demented **(b)** Moderate Demented **(c)** Non-Demented **(d)** Very Mild Demented.

The dataset used in this study is a publicly available MRI dataset sourced from Kaggle, titled the “Augmented Alzheimer MRI Dataset” (22). It comprises a total of 33,984 2D T1-weighted MRI slice images, not full 3D volumes, evenly divided among four diagnostic categories: Mild Demented, Moderate Demented, Non-Demented, and Very Mild Demented as shown in the Figure 2, the images are saved in JPEG format and have undergone data augmentation and applied solely to the training set to enhance diversity and prevent overfitting. The validation and test sets were left unaltered to ensure unbiased evaluation and preprocessing by the original dataset providers and represent 2D slices extracted from volumetric MRI scans. The dataset does not contain subject-level metadata such as age, gender, imaging protocol, or acquisition parameters. Due to the absence of subject identifiers, the dataset was split at the image level rather than the patient level. As a result, adjacent slices from the same volume may exist across training, validation, and test sets, potentially introducing correlation-based bias. The images were divided into training (80%), validation (10%), and testing (10%) subsets, corresponding to 27,188, 3,397, and 3,399 images, respectively. Due to the absence of patient identifiers, the split was performed at the image level, and this limitation is acknowledged as a potential source of correlation bias (23). It is important to note that this dataset includes images that were augmented by the dataset provider prior to release. Therefore, it is most appropriate for use in training and internal evaluation. The lack of access to original, non-augmented scans limits the dataset's suitability for external validation or generalization studies.

The original dataset does not include metadata regarding MRI acquisition protocols, sequence parameters, scanner types, or image reconstruction software, and thus, such details could not be reported in this study. It is important to note that this dataset includes images that were augmented by the dataset provider prior to release. Therefore, it is most appropriate for use in training and internal evaluation. The lack of access to original, non-augmented scans limits the dataset's suitability for external validation or generalization studies. Further, no documentation regarding ethics approval, patient consent, or institutional data sourcing is available for this dataset, and its origin cannot be independently verified. The distribution of the classes is tabulated as follows in Table 2.

**Table 2:** Class wise dataset distribution.

**Dataset**	**No. of images in ‘Mild Demented' class**	**No. of images in ‘Moderate Demented' class**	**No. of images in ‘Non-Demented' class**	**No. of images in ‘Very Mild' class**	**Total images**
Training	6,797	6,797	6,797	6,797	27,188
Validation	850	850	850	850	3,400
Testing	850	850	850	850	3,400
Total	8,497	8,497	8,497	8,497	33,988

### 3.2 Preprocessing

**Figure 3:**
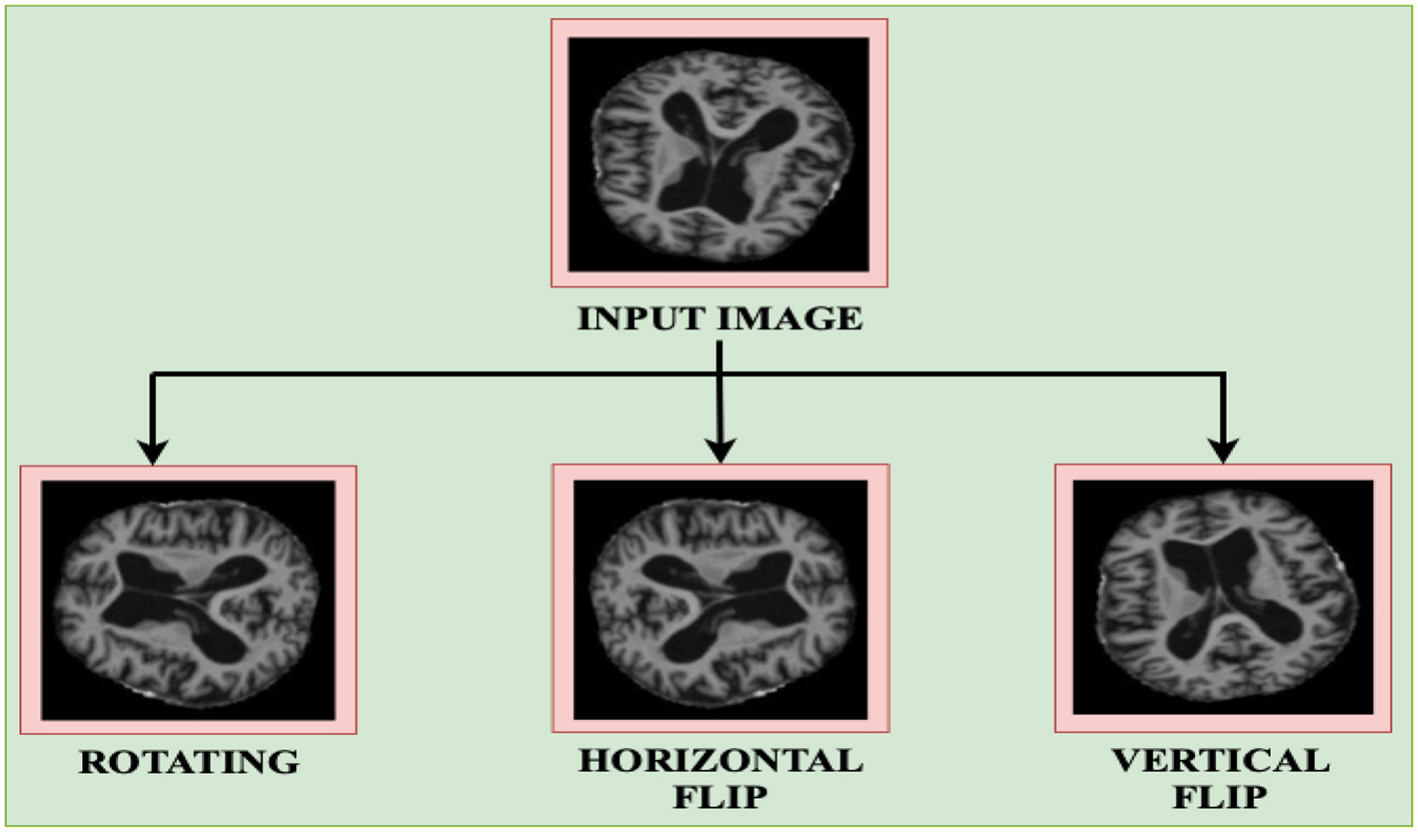
Data augmentation techniques.

In this paper, data preprocessing is found to be a fundamental step in enhancing the machine learning outcomes especially in classifying Alzheimer diseases using MRI scans. Because of the variations witnessed in the quality of images and the small differences in the brain boundaries some preprocessing techniques are very essential to improve the input images (24). First, a process of image normalization is conducted so that the pixel values range from 0 to 1 to reduce possible deviations due to image sizes. Although no explicit denoising or contrast enhancement was applied, several data augmentation techniques were used to enhance the training data and improve model robustness. These included random rotations, zooming, flipping, and brightness variation. All images were resized to 224 × 224 pixels and normalized to a pixel intensity range of [0, 1] before being fed into the models. This makes the model generalized better and also relieves it from overfitting (25). All these preprocessing steps serve to enhance the quality of data put into the ensemble model for the correct identification of Alzheimer's stages (26).

a.) **Normalization:** normalization is the task of adjusting the range of pixel intensities of an image to a standard range, often the interval [0:1]. The most common method is min-max normalization, which can be expressed mathematically as given in the Equation 1 below:
  (1) $$\begin{array}{l} x_{norm}=x-x_{min\frac{x-x_{min}}{x_{-\;x_{min}\;}}} \end{array}$$
  where *x*_*norm*_ is the normalized portion of the pixel value, *x* is the actual pixel value, and *x*_*min*_ is the minimum value of the pixel in the picture, *x*_*max*_ is the maximum value of the pixel in the picture.
b.) **Resizing:** resizing is the process of moving each pixel of an image to a new location in relation to desired width and height of the targeted image. If (*W*_*in*_, *H*_*in*_) is the width and height of the original image and (*W*_*out*_, *H*_*out*_) is the width and height of the resized image. While maintaining the spatial relationships. If (*W*_*in*_, *H*_*in*_) be the width and height of the original image and (*W*_*out*_, *H*_*out*_) be the width and height of the resized image. To standardize input dimensions for model training, each 2D MRI slice was resized to 224 × 224 pixels using bilinear interpolation. This resizing adjusted the number of image pixels but did not account for physical voxel dimensions, which could not be preserved due to the absence of spatial resolution metadata in the JPEG-formatted dataset. So, the scaling factors for width and height are computed as in Equation 2 below:
  (2) $$\begin{array}{l} s_{w\;}=\;\frac{W_{out}}{W_{in}}\;,\;s_{h\;}=\;\frac{H_{out}}{H_{in}} \end{array}$$
c.) **Data augmentation:** data augmentation involves applying various operations on the existing dataset in order to create an enlarged and diversified set in order to improve generalization. It features different augmentations like rotation, scaling, shifting, flipping among others as shown in Figure 3. Rotating an image by angle θ is given by the formula as shown in the Equation 3 below.
  (3) $$\begin{array}{l} \left[x^{\prime }y^{\prime }\right]=\left[cos\theta -sin\theta \;sin\theta cos\theta \right]\left[x\;y\;\right] \end{array}$$
  where, the coordinate position of the original raster image pixel is designated by (*x, y*) and that of the new position is by (*x*′, *y*′) and the angle of rotation is θ in radians. Horizontal flipping reflects an image across the vertical axis. This transformation can be mathematically represented by reversing the *x*-coordinate of each pixel as in Equation 4:
  (4) $$\begin{array}{l} x^{\prime }=-x,\;y^{\prime }=y \end{array}$$

This augmentation is particularly useful in medical imaging to introduce left–right symmetry, thereby improving the model's robustness to orientation variance.

Vertical flipping reflects the image across the horizontal axis and is represented as in Equation 5 below:


(5) $$\begin{array}{l} x^{\prime }=x,\;y^{\prime }=-y \end{array}$$

This operation helps simulate top–bottom inversion, further enhancing the model's ability to learn invariant spatial features, especially when orientation does not impact diagnostic relevance.

### 3.3 Model building

Two architectures of deep learning models, the ResNet50 and EfficientNet-B7 that form the basis of the ensemble model are generated by this method. Each model is established meticulously to construct components of MRI images essential for satisfying classification exclusively.

#### 3.3.1 ResNet-50

**Figure 4:**
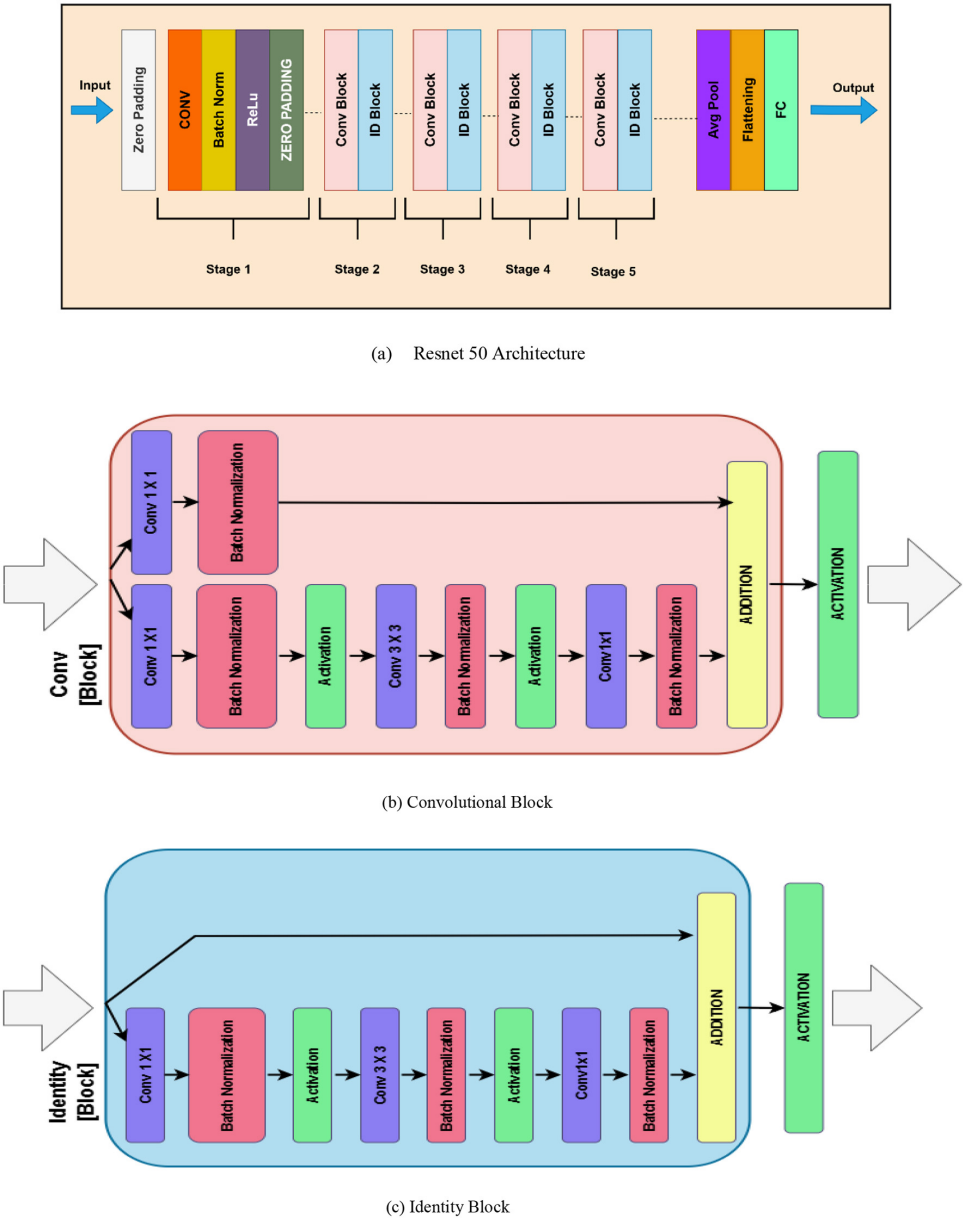
Resnet 50 **(a)** Resnet 50 architecture **(b)** Convolutional block **(c)** Identity block.

ResNet-50 consists of 50 layers, including convolutional layers, pooling layers, batch normalization (BN), and fully connected layers, as illustrated in Figure 4a. ResNet' s principal invention is the residual block; this essential function is a “shortcut” or direct pathway that sends the input to the layer through to the output. This allows the model they base to skip certain layers and decrease the gradient disappearance problem in very deep networks (27). These shortcut connections help the network retain accuracies of deeper models possible without crossing the degradation issue by “jumping” other layers. The recognized blocks of architecture include the pooling layers, batch normalization, ReLU activation functions, and convolutional layers in sequence is given mathematically by Equation 6.


(6) $$\begin{array}{l} y=F\left(x,\left\{w_{i\;}\right\}\right)+x \end{array}$$

Here *x* is the input to the residual block, *y* is the output, *F*(*x*{*w*_*i*_}) is the function that is applied on the input *x*. The last layer of classification produces output *z*_*resnet*_ as described below in Equation 7 after passing through the network.


(7) $$\begin{array}{l} y_{resnet}=softmax\left(W_{resnet}\times Y_{global}+b_{resnet}\right) \end{array}$$

Here, *W*_*resnet*_ and *b*_*resnet*_ are the weights and biases of the dense layer, and *Y*_*global*_ is the output from the global average pooling layer.

The convolutional block from ResNet-50 as illustrated in the Figure 4b, is a deep convolutional neural network that aids in the vanishing gradient problem through the element of residual learning. This block was implemented with the intent of being used to extract features while still allowing deeper networks to learn. The convolutional block includes three types of convolutional layers implemented in a sequence. The first layer is a 1 × 1 convolution that decreases the dimension of the input feature maps in order to lessen computational cost Stage 3: technology 3. The second one is another convolution layer with size 3 × 3 to cover spatial connections and explicit features. The third layer is another 1 × 1 convolution to get back to the original dimensions of the feature maps. After each convolution there is normalization to make the training process faster and more stable, as well as using activation function (ReLU). The feature that is unique to the convolutional block is the projection shortcut connection, which uses 1 × 1 convolution to bring the dimensions of the input to match that of the processed features. This makes some sense as it actually establishes compatibility for the element-wise addition on the shortcut and the convoluted feature maps. Then a feedback layer addition is applied, and finally has the activation function to get the output. This design makes it possible for ResNet-50 to learn initially both low level and high-level features in deep networks.

In addition, an identity block in ResNet-50 as depicted in Figure 4c is an essential building block aimed at transferring features well through deep architectures. As it will be seen, the identity block retains the input dimensions since it uses a skip connection that feeds the input directly to the output without any change of dimension. This helps in making the model fast and stable while processing in the later stage of the training. The identity block contains three layers of convolution. The first is a 1 × 11 times one convolution layer that is aimed at the dimensionality of the input feature maps. This is succeeded by a 3 × 33 times three convolution which extracts spatial features and patterns, and one more 1 × 11 times one convolution which brings back dimensionality. Each convolutional layer is associated with batch normalization to update the activation for acceleration of convergence as well as activation function like ReLU. The key feature of the identity block is that the input directly connects to the output without passing through the convolutional layers by adding the input feature maps with the corresponding feature maps after passing through the network. After this addition there is an activation function to produce the output. The added identity block makes ResNet-50 deepen this network while allowing it to maintain the hoisting of features and avoid the vanishing gradient issue, making it a great architecture for acquiring features.

#### 3.3.2 Efficient Net

**Figure 5:**
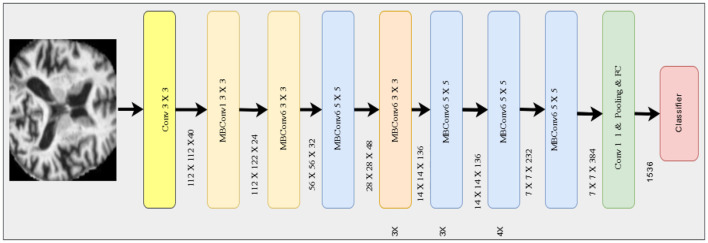
Efficient Net B3 architecture.

Based on a compound scaling coefficient, Efficient Net aims to optimize at the same time depth, width and the resolution according to a parameter Ø that represents a family of models. EfficientNet-B3 is one particular network in the Efficient Net series of models and, as with all models in this series, this network enforces a balance between these three aspects to yield decent compromise between model complexity, model accuracy, and compute requirements (26). The scaling is governed by Equation 8:


(8) $$\begin{array}{l} d=\alpha^{\phi },w=\beta^{\phi },r=\gamma^{\phi } \end{array}$$

where *d, w*, and *r* are the network's depth, width, and resolution, respectively and where α, β, and γ are parameters. The output of EfficientNet-B7, after global average pooling, is shown in Equation 9:


(9) $$\begin{array}{l} y_{efficientnet}=softmax\left(W_{efficientnet}\times f_{global}+b_{efficientnet}\right) \end{array}$$

where *f*_*global*_ is the feature vector, and *W*_*efficientnet*_, *b*_*efficientnet*_ are the weights and the biases of the dense layer. The architecture of EfficientNet-B7 demands for many important components: from original input, features are extracted by convolutional layers to improve gradient flow and achieve batch normalization and the Swish activation function. The Figure 5 shows the architecture of Efficient Net B3.

#### 3.3.3 Ensemble model architecture

**Figure 6:**
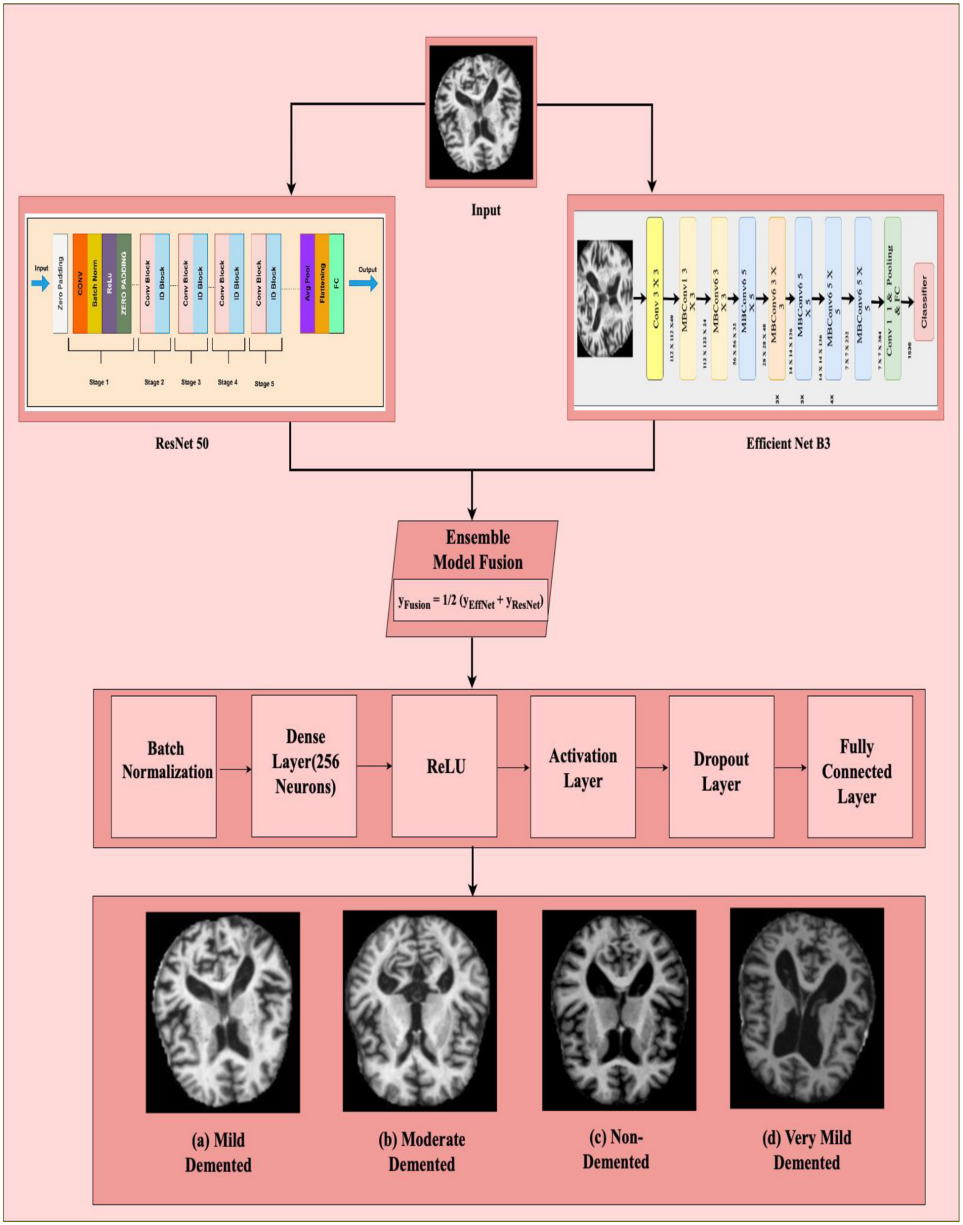
Ensemble model architecture. The framework consists of: (1) Input brain MRI image; (2) Feature extraction using ResNet-50 and EfficientNet-B3; (3) Ensemble model fusion, where outputs of ResNet-50 and EfficientNet-B3 are combined; (4) Classification head composed of Batch Normalization, Dense Layer (256 neurons), ReLU, Activation Layer, Dropout Layer, and Fully Connected Layer; and (5) Final classification into four categories: **(a)** Mild Demented, **(b)** Moderate Demented, **(c)** Non-Demented, and **(d)** Very Mild Demented.

In the proposed ensemble model, ResNet-50 and EfficientNet-B3 were trained independently using the same training dataset to classify MRI slices into four Alzheimer's disease stages. During inference, both models generate probability scores for each class through softmax layers, and these outputs are combined using a soft voting approach by simply averaging the predictions. This fusion allows the ensemble to benefit from the complementary strengths of both networks: EfficientNet-B3 offers high efficiency with fewer parameters, while ResNet-50 contributes deep hierarchical feature extraction through residual learning. To stabilize training and reduce internal covariate shift, batch normalization is applied to the fused features, followed by a dense layer with 256 neurons and ReLU activation for non-linearity. Regularization techniques, including both L1 and L2 penalties, are applied to prevent overfitting, and a dropout layer is used to further improve generalization. The final classification is performed through a fully connected layer that maps the processed features to class probabilities. The model is trained using categorical cross-entropy loss, which evaluates the difference between predicted and true class labels. Overall, this ensemble design enhances diagnostic performance by combining the robustness of two diverse deep learning architectures as in the Figure 6. Rather than assigning weighted average or performing any other operation, the outputs from both the models are then simply averaged as they have been observed to complement each other. EfficientNet-B3 gives state of the art efficient feature representation using fewer number of parameters compared to ResNet-50 which offers strong hierarchical feature representation due to its residual learning (11). The combined output fusion is computed as shown in Equation 10 where *y*_*EffNet*_is the final prediction of Efficient B3 and *y*_*ResNet*_is the final prediction of Resnet 50.


(10) $$\begin{array}{l} y_{Fusion\;}=\frac{1}{2}.\left(y_{EffNet}+\;y_{ResNet}\right) \end{array}$$

This particular fusion strategy also ensures that both models contribute equally enough to the ensemble so that generalization over the various patterns across images will be well-captured.

Batch normalization (BN) is then employed on the fused features to stabilize and enhance the speed of the whole training process by normalizing the outcomes. The normalized feature vector ŷ is computed as in Equation 11:


(11) $$\begin{array}{l} \hat{y}=\;\frac{y_{Fusion-\;\mu }}{\sqrt{\sigma^{2\;}}+\epsilon } \end{array}$$

where μ and σ^2^ are the estimate of average of the batch, and variance of the batch respectively and ϵ is a small constant value so as to avoid division by zero. Trainable scaling (γ) and shifting (β) parameters further refine the normalized features by using the Equation 12:


(12) $$\begin{array}{l} y^{\prime }=\gamma .\hat{y}+\beta \end{array}$$

This step reduces the covariate shift problem within the organization's internal environment, meaning that there is a more stable distribution of the particular features through the layers. The features being batch normalized are then fed through a dense layer with 256 output neurons. This layer applies a linear transformation followed by a ReLU activation for non-linearity as shown in Equation 13:


(13) $$\begin{array}{l} z=ReLU\left(W.\;y^{\prime }+b\right) \end{array}$$

where, *W* is Weight matrix, *b* is Biase vector and ReLU(a) = max (0, a) To prevent overfitting, L1 and L2 regularization terms are added to the loss function, penalizing large weights as in Equation 14:


(14) $$\begin{array}{l} Regularization\;Loss=\lambda_{1.}{∥\;W∥}_{1}+\lambda_{2.}{∥\;W∥}_{2}^{2} \end{array}$$

Also, Dropout layer which drops out neurons with the probability *p* is implemented to increase the ability of generalization of the model. The last fully connected layer adopts the SoftMax function in order to convert the distilled features to probabilistic outcomes reflecting the number of categories of the output. For each class *k*, the output probability *y*_*k*_ is given by Equation 15:


(15) $$\begin{array}{l} y_{k}=\frac{exp\;exp\;\left(z_{k}\right)\;}{\sum_{j=1}^{C}=exp(z_{j})} \end{array}$$

where *C* represents the number of classes, while *z*_*k*_ is the logit for class *k*. The model is trained using categorical cross-entropy loss, minimizing the divergence between true labels *y*_*i, k*_ and predicted probabilities *y*_(i, k)_ as in Equation 16


(16) $$\begin{array}{l} L=\;-\frac{1}{N}\;\sum_{i=1}^{N}\;\sum_{k=1}^{C}\;y_{i,k\;}log\left({\hat{y}}_{i,k\;}\right) \end{array}$$

All the enhancement methods used in the proposed ensemble model, namely, feature fusion, normalization, dense layers, and regularization, make it highly capable to perform well in the classification of Alzheimer's disease. Using EfficientNet-B3 and ResNet-50, this approach offers significant capabilities for the early diagnosis, which further outperform the outcomes of separate models with higher accuracy and their generality. A dropout layer is applied after the ReLU-activated dense layer and before the final classification layer to reduce overfitting and improve generalization.

#### 3.3.4 Hyperparameter details

To ensure optimal model performance and training stability, a carefully selected and tuned range of hyperparameters for both ResNet-50 and EfficientNet-B3 models used in the ensemble (11). These parameters were chosen based on preliminary experimentation and established best practices in deep learning for medical imaging. Key hyperparameters include the choice of optimizer, learning rate, batch size, number of training epochs. A detailed summary of the hyperparameters used in this study is provided in Table 3. These settings were consistent across both models to ensure fairness and effective ensemble integration. The models were developed using Python 3.8 with the TensorFlow 2.9 and Keras libraries. Additional preprocessing and evaluation were performed using NumPy, OpenCV, scikit-learn, and Matplotlib.

**Table 3:** Training hyperparameters.

**Hyperparameter details**	**Value/description**
Optimizer	Adam
Learning rate	0.0001
Loss function	Categorical cross entropy
Batch size	32
Number of epochs	10
Input image size	224 × 224 × 3
Dropout rate	0.5
Data split ratio	80% Training 10% Validation 10% Testing
Data augmentation	Rotation, Zooming
Framework used	Python 3.8, TensorFlow 2.9, Keras, OpenCV, NumPy, Matplotlib

## 4 Results

This section presents the experimental results obtained from evaluating the proposed ensemble model comprising ResNet-50 and EfficientNet-B3 on the Alzheimer's MRI classification task. The model's performance was assessed using standard evaluation metrics, including accuracy, precision, recall, and F1-score across four Alzheimer's disease stages: Non-Demented, Very Mild Demented, Mild Demented, and Moderate Demented. The results demonstrate that the ensemble approach outperforms individual models in terms of both classification accuracy and generalization capability. Detailed comparisons, confusion matrices, and performance tables are provided to illustrate the effectiveness of the proposed method and support its potential for clinical deployment in diagnostic workflows.

### 4.1 Evaluation parameters

An evaluation parameter is a measure by which the performance, efficiency or effectiveness of a model, process, or system can be judged. Such parameters are commonly applied in different areas including machine learning, statistics, finance and engineering.

a) Accuracy: accuracy in multi-class classification is defined as the ratio of correctly predicted samples to the total number of samples across all classes. It measures the overall effectiveness of the model in assigning the correct label to each input as in Equation 17 below:


(17) $$\begin{array}{l} Accuracy=\frac{No.\;of\;correct\;predictions}{Total\;No.\;of\;predictions}=\frac{\sum i=1CTPi}{N} \end{array}$$

Where *TPi* = True Positives for class *i, C* = Total number of classes, *N* = Total number of samples, where *i* can be any class out of four classes of Alzheimer.

b) Precision: precision measures the proportion of correct positive predictions for each class out of all predictions made for that class. It indicates how many of the predicted instances for a specific class are actually correct. Precision is presented by the formula of precision expressed in Equation 18 below:


(18) $$\begin{array}{l} Precision_{i}=\frac{TPi}{TPi+FPi} \end{array}$$

c) Recall: recall, also known as sensitivity, measures the proportion of actual positives that were correctly identified for each class. It shows how well the model captures the true instances of each class. The formula of precision is expressed below in Equation 19 below:


(19) $$\begin{array}{l} Recall_{i}=\frac{TPi}{TPi+FNi} \end{array}$$

Where *FN*_*i*_ is false negative for class *i*.

d) F1-Score: the F1-score is the harmonic mean of precision and recall for each class. It balances the trade-off between precision and recall, especially useful when classes are imbalanced. The F1-score is calculated as shown in Equation 20:


(20) $$\begin{array}{l} {F1}_{i}-Score=2\;\times \;\frac{\left(Precision_{i}\;\times \;Recall_{i}\right)}{\left(Precision_{i}+\;Recall_{i}\right)} \end{array}$$

### 4.2 Training and validation results

Comparative analysis of performance was conducted between ResNet-50 and EfficientNet-B3 during their training and validation stages. Two different computational frameworks trained against a predefined dataset to evaluate their performance by calculating their accuracy and precision during validation with recall and F1-score metrics achieved alongside AUC-ROC value evaluations. The feature extraction abilities of ResNet-50 were excellent but required precision adjustments through fine-tuning to reach its best levels of operation. The efficient scaling of EfficientNet-B3 produced superior accuracy results while maintaining better generalization capabilities. The validation results showed that EfficientNet-B3 demonstrated better performance than ResNet-50 models primarily because of its superior structural design. Background inference speed retained similarity between ResNet-50 and other comparison models. A decision between the two systems depends on whether applications prioritize accuracy or computational speed. The model was evaluated using multi-class performance metrics, including overall accuracy, precision, recall, and F1-score. These metrics were calculated for each of the four classes individually and macro-averaged to provide an overall assessment.

#### 4.2.1 Training and validation results of efficient net B3

**Figure 7:**
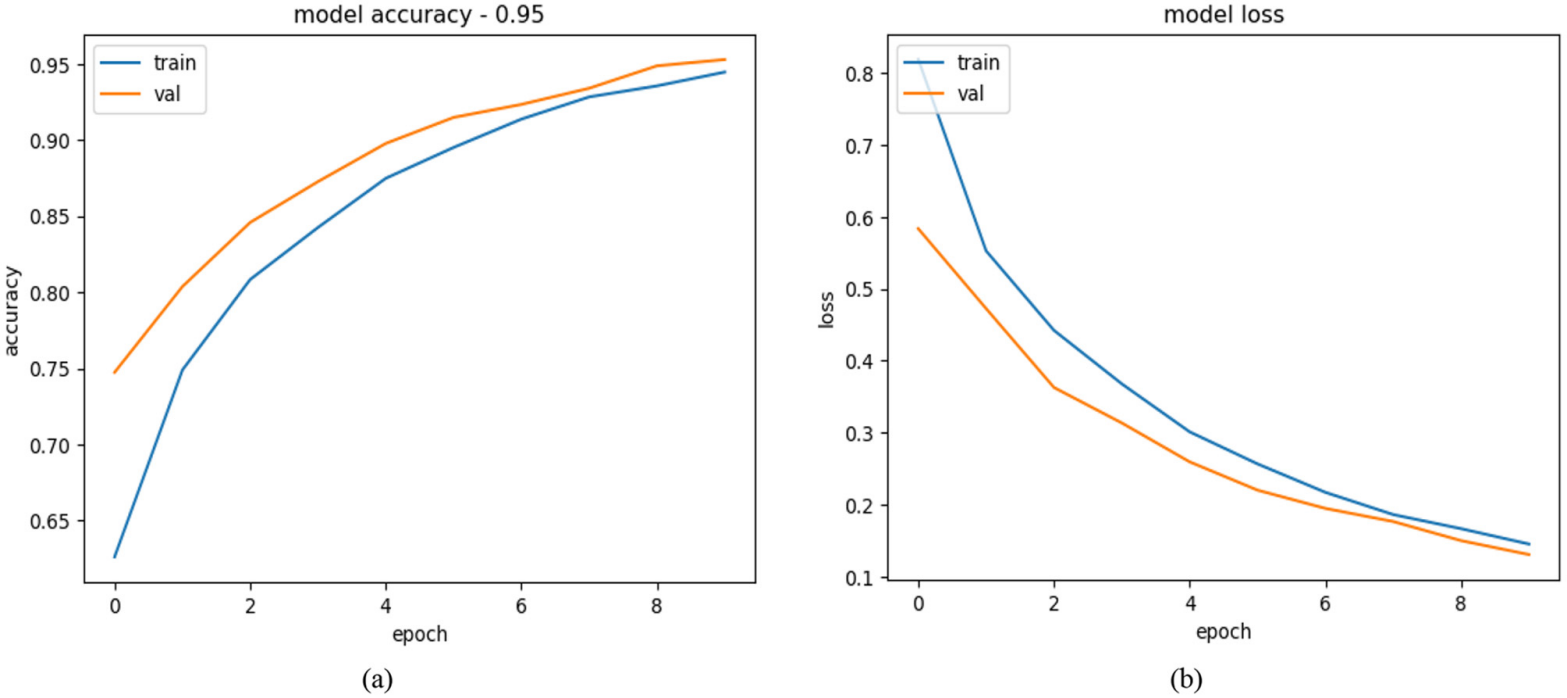
**(a)** Model accuracy **(b)** model loss of efficient net B3.

Performance trends from the EfficientNetB3 based Alzheimer's disease detection model can be found in the depicted accuracy and loss data plots. The deployment of 10 epochs throughout training yielded positive results which appeared in both training and validation metrics. Both training and validation data show continuous performance improvements throughout the epochs according to the accuracy plot displayed on the left. The initial training accuracy level was ~65% before reaching near 95% stability. The generalization capacity becomes evident through the validation accuracy which shows a start value higher than training accuracy and converges to 95%. The models training and validation accuracy graphs remain close together which means the model avoids major overfitting problems. Training along with validation loss shows continuous reduction throughout the overall training process according to the loss plot. Training losses initiate at 0.7 but continuously decrease and settle near 0.1 by the end of training (28). The validation loss chain shows a downward movement which starts underneath the training loss mark then reaches similar value terminals at epoch completion. The model's robust structure receives additional confirmation through the parallel changes observed in validation and training loss metrics. Effective learning and generalization abilities stand out in the EfficientNetB3 architecture when used for Alzheimer's disease detection based on its metric convergence performance. The balanced performance of training and validation curves demonstrates that the model effectively extracts significant data features while avoiding overfitting which demonstrates its practical utility in clinical diagnostics settings. All performance metrics are displayed through the graphs presented in Figure 7.

#### 4.2.2 Training and validation results of ResNet 50

**Figure 8:**
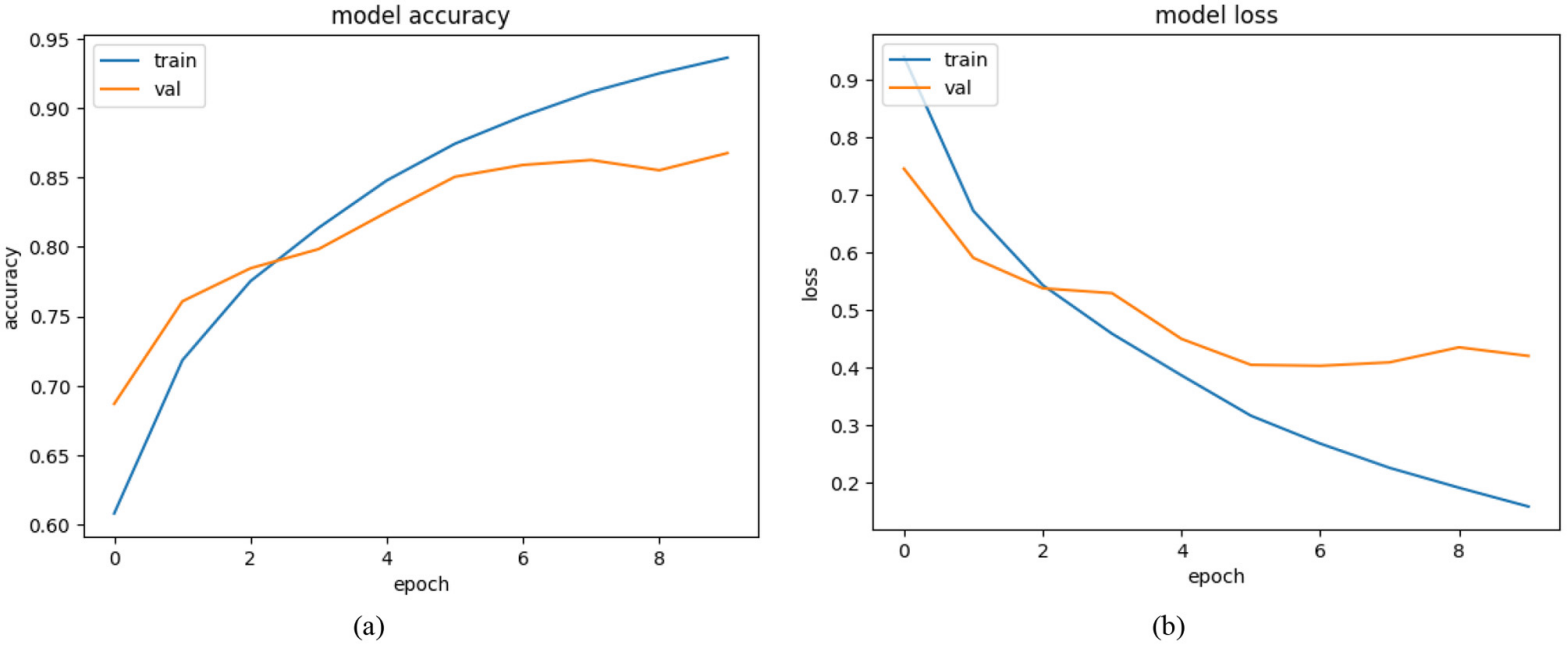
**(a)** Model accuracy **(b)** model loss of Resnet 50.

Multiple plots show the performance metrics between training data accuracy and validation data accuracy alongside training data loss and validation data loss when using ResNet-50 for Alzheimer's disease prediction. The training process required 10 epochs toward model evolution yet the performance metrics showed some separateness between training and validation results. The accuracy graph (left) demonstrates that model training accuracy gradually improved from 60% to a nearly 95% level throughout ten epochs. Initially the validation accuracy started at ~70% then climbed to reach nearly 87% values. Beyond the fifth epoch the validation accuracy demonstrates unstable patterns which could be explained by overfitting and changes found within the validation dataset. The decreasing trend on loss data demonstrates successful learning between training data along with validation data. Training loss begins at 0.9 before reaching 0.2 only after completing the training period. From its starting point at 0.8 the validation loss gradually lowers until reaching a minimum of 0.4 at epoch five. Beyond epoch 5 the validation loss exhibits a tiny upward trend because the model effectively performs on training data however, it misses essential patterns needed for unseen input recognition (29). Throughout the later part of training the separation between validation and training performance metrics demonstrates that ResNet-50 successfully grasps patterns from the data although it needs further development for generalized results. Early stopping alongside data augmentation and standard techniques for regularization offer potential solutions to reduce overfitting. The ResNet-50 model shows promise for Alzheimer's disease detection capabilities through its excellent training accuracy results and fair validation performance potential that creates opportunities for future clinical diagnostic applications. All performance metrics have their graphical representations displayed in Figure 8.

#### 4.2.3 Training and validation results of proposed ensemble model

**Figure 9:**
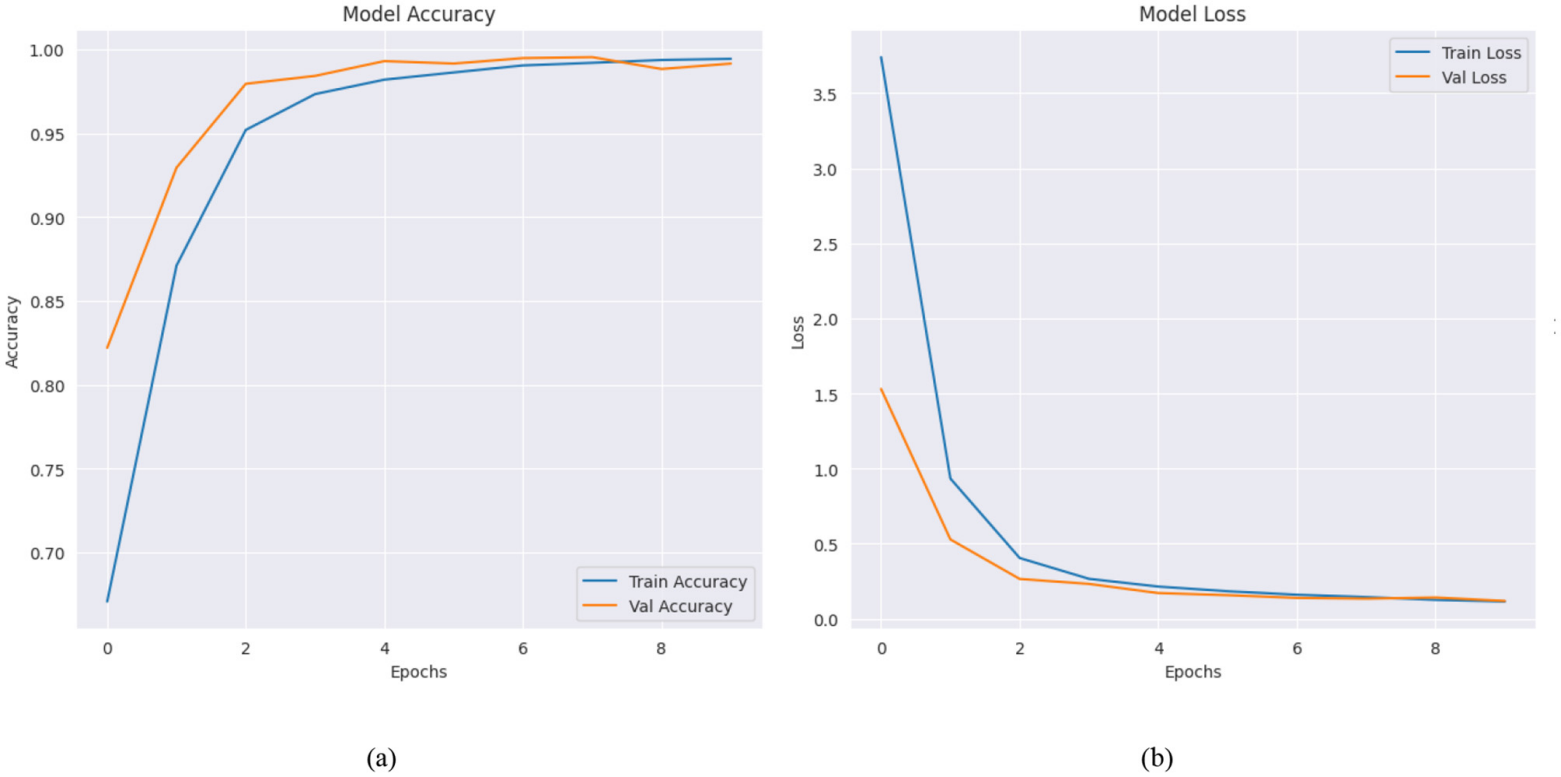
**(a)** Model accuracy **(b)** model loss of ensemble model.

These graphic displays show how an ensemble with ResNet-50 and EfficientNetB3 models detects Alzheimer's disease throughout 10 training cycles. The left graph shows accuracy performance which demonstrates exceptional model behavior through rapid improvement of training and validation accuracy toward perfect scores. The model establishes an initial training accuracy baseline at 70% which evolves into 100% accuracy during the fourth epoch then maintains peak performance for the remaining epochs. The baseline validation accuracy sits at 85% during the initial stage after which it establishes perfect synchronization with training accuracy throughout subsequent epochs. The coaches' curves align perfectly which demonstrates the model will generalize successfully and avoids excessive overfitting behavior. A loss plot analysis reveals that both training and validation loss decrease sharply in initial epochs to stabilize at low levels. Training loss displays initial values of about 3.5 that diminish rapidly to less than one unit during epoch 5 then settles down at that minimum value point. Validation loss displays a parallel reduction pattern which starts near 2.5 before decreasing under 0.5 during epoch 4 while training loss tracks closely in subsequent epochs (30). The parallel development of accurate results and low loss data points demonstrates the sturdy characteristics of the ensemble model system. The ensemble methodology uses ResNet-50 and EfficientNetB0 to extract complementary functionality which delivers outstanding results for Alzheimer's disease diagnosis. The model demonstrates accurate pattern recognition in the data through quick criterion alignment and data metric convergence without producing overfitting issues. The ensemble approach demonstrates potential utility as a dependable medical diagnostic instrument since it delivers accurate results alongside sharp dataset generalization abilities. All performance metrics are displayed graphically in Figure 9.

#### 4.2.4 Comparison results of ensemble model, EfficientNet-B3 and ResNet50

The performance metrics for multiple deep learning models across ten epochs are shown in Table 4 where training accuracy and validation accuracy and validation F1-score are evaluated. Scientists apply equivalent deep learning technologies from this domain to detect Alzheimer's disease through MRI medical imaging. The progressive neurodegenerative psychiatric condition Alzheimer's disease leads to cognitive decline so it requires early diagnosis to deliver effective therapeutic measures. The diagnostic systems built with CAD capabilities utilize EfficientNet-B3 along with ResNet50 and ensemble models as they demonstrate exceptional accuracy in image recognition tasks. The training and validation accuracy of both EfficientNet-B3 and ResNet50 increase through epochs and the ensemble model exceeds the performance of each model individually. All performance metrics, including accuracy, precision, recall, and F1-score, were calculated in a multi-class setting across four classes. Per-class metrics were computed and macro-averaged to summarize overall model performance. Ensemble learning proves beneficial because diverse model combinations increase generalization ability which then produces superior diagnostic results. Deep learning models trained with Alzheimer's Disease Neuroimaging Initiative (ADNI) medical images demonstrate potential for Alzheimer's disease detection applications. The EfficientNet-B3 model demonstrates top capability in extracting MRI scan features followed by ResNet50 which automatically adjusts training depths to overcome vanishing-gradient difficulties by using its residual learning method. The ensemble model's high performing results indicate that using multiple architectures enhances detection accuracy for early-stage Alzheimer's disease. The F1-score acts as a vital tool for medical researchers because it evaluates model performance specifically during assessment of diagnosis systems which operate on imbalanced datasets primarily featuring underrepresented early-stage and mild Alzheimer's cases. Analysis of the F1-score values shows that the ensemble model maintains its superior performance throughout all epochs while achieving optimal precision and recall ratings. Morocco's scientific research benefits from F1-score accuracy which strives to improve disease detection at both non-diseased and diseased case levels thereby supporting clinical tools development. Model learning effectiveness and generalization ability increase concurrently with validation accuracy across epochs which proves fundamental when applying medical approaches to real-world situations. Deep learning algorithms with similar models from the table enable researchers to create dependable CAD systems which benefit neurologists through improved Alzheimer's disease diagnosis accuracy. The diagnostic accuracy can be improved by two techniques: domain-specific transfer learning fine-tuning and additional multimodal data analysis. Deep learning demonstrates its critical role in disease detection through the data trends presented in the table. Researchers implementing these technologies in Alzheimer's detection will achieve early diagnosis while enabling faster interventions that ultimately lead to better patient results. The Table 4 below shows the comparison of Resnet 50, Efficient Net B3, and ensemble model.

**Table 4:** Comparison of ResNet-50, EfficientNet-B3, and Ensemble model.

**Epoch**	**Model**	**Training accuracy**	**Validation accuracy**	**Validation F1-score**
1	EfficientNet-B3	0.6261	0.7473	0.5482
	ResNet50	0.608	0.687	0.686
	Ensemble model	0.6707	0.822	0.8365
2	EfficientNet-B3	0.7489	0.8037	0.6947
	ResNet50	0.7184	0.7608	0.758
	Ensemble model	0.8709	0.9294	0.9355
3	EfficientNet-B3	0.8083	0.8458	0.7797
	ResNet50	0.7754	0.7846	0.784
	Ensemble model	0.9519	0.9794	0.9809
4	EfficientNet-B3	0.8425	0.8726	0.8349
	ResNet50	0.8138	0.7985	0.798
	Ensemble model	0.9733	0.9841	0.9854
5	EfficientNet-B3	0.8748	0.8977	0.8889
	ResNet50	0.8479	0.8249	0.824
	Ensemble model	0.9819	0.9929	0.9935
6	EfficientNet-B3	0.8951	0.9148	0.9124
	ResNet50	0.8744	0.8505	0.85
	Ensemble model	0.9862	0.9915	0.9922
7	EfficientNet-B3	0.9137	0.9233	0.9201
	ResNet50	0.8942	0.8591	0.859
	Ensemble model	0.9904	0.9947	0.9951
8	EfficientNet-B3	0.9283	0.934	0.9311
	ResNet50	0.9116	0.8626	0.862
	Ensemble model	0.9919	0.9953	0.9957
9	EfficientNet-B3	0.9355	0.9487	0.946
	ResNet50	0.925	0.8553	0.855
	Ensemble model	0.9936	0.9882	0.9891
10	EfficientNet-B3	0.9446	0.9528	0.9504
	ResNet50	0.9363	0.8676	0.868
	Ensemble model	0.9943	0.9915	0.9922

### 4.3 Testing results

Real-world testing of ResNet-50 and EfficientNet-B3 produced evaluation results. The superior generalization capabilities of EfficientNet-B3 became evident through improved accuracy and precision together with enhanced recall. The model was superior to ResNet-50 in recognizing minimal patterns while producing fewer mistakes. The real-time applications could benefit from the ResNet-50 model because it delivers inference operations at a faster pace. The scoring system emphasized EfficientNet-B3 as the best model in discrimination capability assessment. The efficiency of ResNet-50 did not reduce its competitive strength unless optimum hyperparameters were used. Two efficient network choices exist: EfficientNet-B3 provides enhanced accuracy while ResNet-50 delivers crucial speed performance for applications. Additional adjustments to model parameters combined with better data preparation will help increase test results from both systems.

#### 4.3.1 Classification results of EcientNet-B3, ResNet50, and ensemble model

The classification report in Table 5 provides a comprehensive breakdown on testing models across four categories by showing accuracy data as well as recall metrics alongside F1-score percentages and class support counts. Our results show the ensemble model based on ResNet50 plus EfficientNet-B3 delivers advanced detection of Alzheimer's disease across all four disease classification levels. The ensemble model executed with ResNet50 and EfficientNet-B3 demonstrated absolute classification precision and recall and F1-score values of 1.00 for detecting Mild Demented, Moderate Demented and Non-Demented cases. The model maintains a precision rate of 0.98 and recall rate of 1.00 when classifying Very Mild Demented images. This produces an F1-score of 0.99. Evaluation shows that when measuring performance separately, the EfficientNet-B3 model produces superior results than ResNet50 because it achieves 0.95 precision compared to 0.87 precision together with 0.95 recall compared to 0.87 recall which generates a superior overall F1-score. The F1-score of EfficientNet-B3 achieves 1.00 in detecting Moderate Demented cases in particular together with strong performance in all present classes. ResNet50 demonstrates reduced performance in identifying Very Mild Demented cases and achieves recall levels of 0.76 thereby affecting its overall classification precision. The coordinating method capitalizes on the individual capabilities of both systems thereby enhancing overall classification performance. The ensemble model demonstrates reliable performance with an overall accuracy rating of 0.9932 which confirms its potential use for automated Alzheimer's disease detection.

**Table 5:** Comparison of various parameters under different models.

**Class**	**Model**	**Precision**	**Recall**	**F1-score**	**Support**
Mild Demented	Ensemble model	1	1	1	896
Moderate Demented		1	1	1	647
Non-Demented		1	1	0.99	960
Very Mild Demented		0.98	1	0.99	896
Mild Demented	ResNet50	0.82	0.92	0.87	896
Moderate Demented		0.99	0.98	0.98	927
Non-Demented		0.84	0.85	0.84	927
Very Mild Demented		0.86	0.76	0.81	907
Mild Demented	Efficient net B3	0.96	0.98	0.97	932
Moderate Demented		0.99	1	1	602
Non-Demented		0.93	0.94	0.94	979
Very Mild Demented		0.94	0.91	0.93	886
Overall accuracy	Ensemble model	0.99	0.99	0.99	3,399
	ResNet50	0.87	0.87	0.87	3,399
	Efficient Net B3	0.95	0.95	0.95	3,399

#### 4.3.2 Confusion matrix of EfficientNet-B3

**Figure 10:**
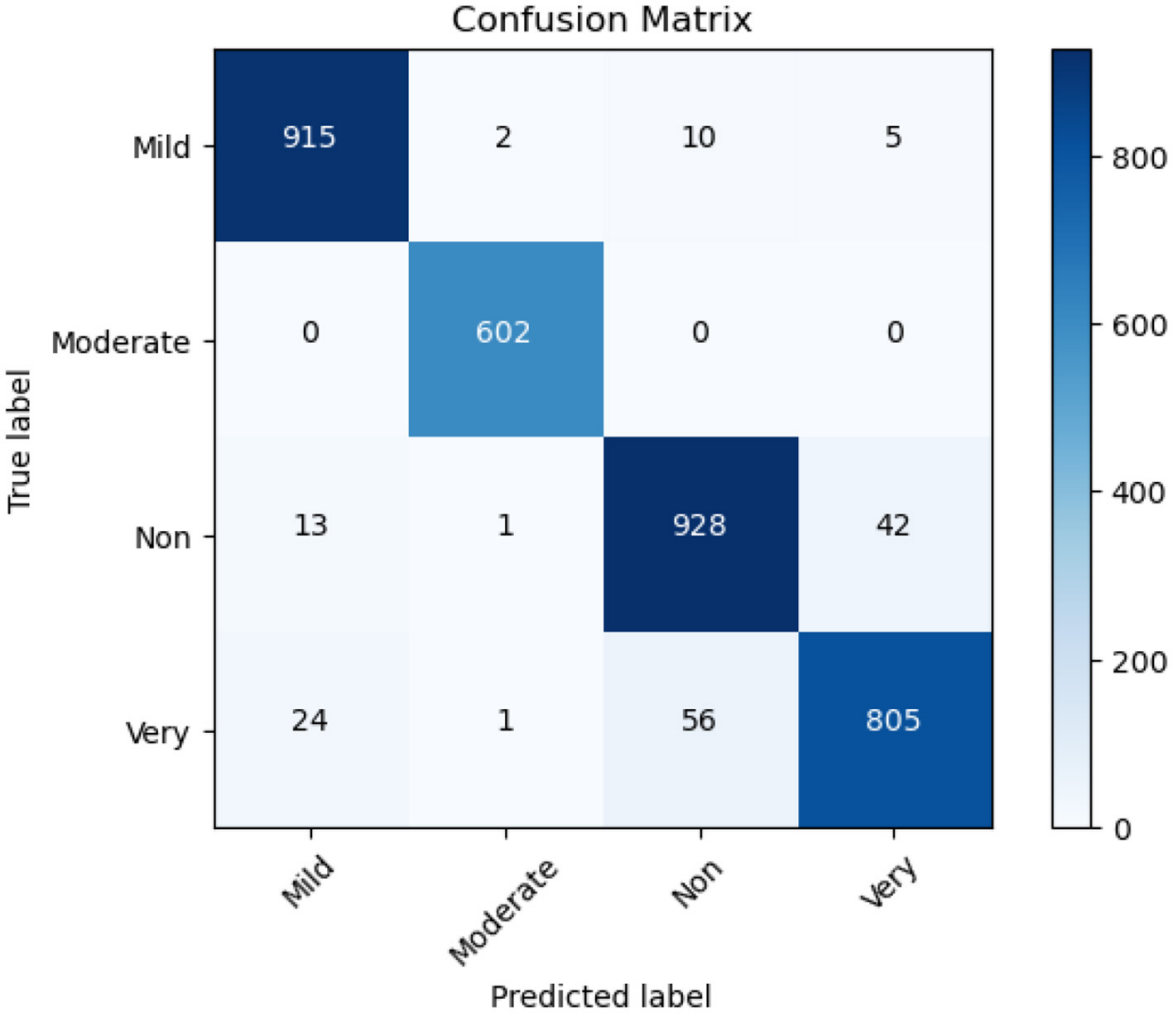
Confusion matrix of Efficient Net B3.

A confusion matrix serves as a performance evaluation tool which enables researchers to evaluate how machine learning models classify different data points. A basic mathematical unit that displays the real classification output with the model prediction output during model analysis. The rows display real-world labeling and the columns deliver model prediction classes. The research invests in studying the confusion matrices obtained from the Ensemble Model alongside ResNet50 and EfficientNet-B3. The confusion matrix in Figure 10 evaluates the EfficientNet-B3 model's performance in classifying Alzheimer's disease stages: Mild, Moderate, Non, and Very. The model demonstrates impressive accuracy by accurately identifying Mild (915 correct) and Moderate (602 correct) cases paired with sparse misdiagnosis occurrences. The identification of non-Alzheimer's international cases proves reliable at 928 while showing some wrong assignments of very severity. Severe cases (805 correct) show occasional confusion with Non-cases (56 misclassified). The successful early and moderate stage differentiation by EfficientNet-B3 needs improvements for better discrimination between severe disease presentations and non-diseased conditions to create accurate tools for clinical diagnosis.

#### 4.3.3 Confusion matrix of ResNet 50

**Figure 11:**
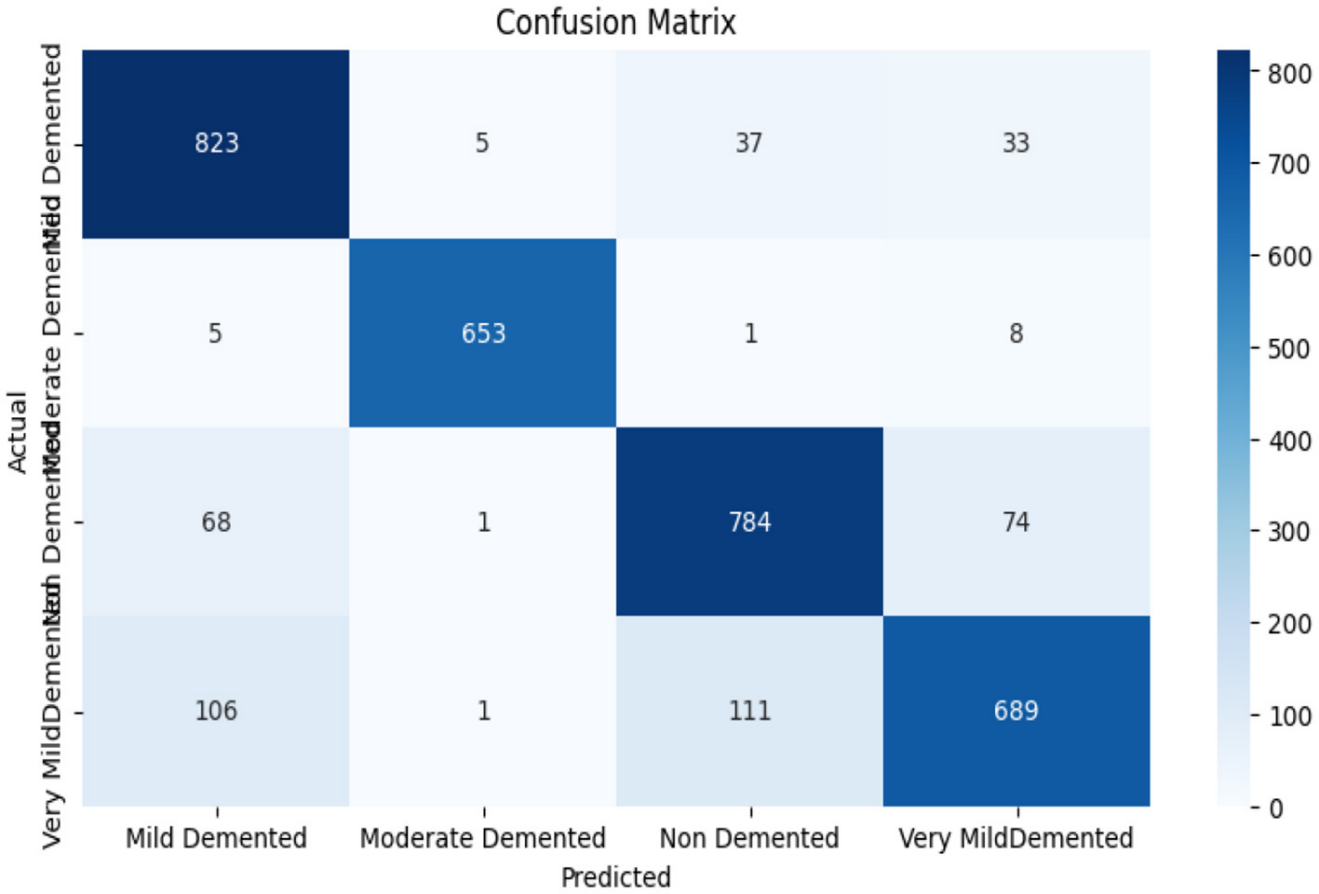
Confusion matrix of ResNet 50.

The Resnet 50 model delivers excellent diagnostic accuracy when distinguishing between Mild Demented and Non-Demented groups since it makes 823 and 784 correct determinations at once. The evaluation shows certain classification errors occur most frequently between Very Mild Demented and Non-Demented categories. Habitat Resnet 50 demonstrates accurate performance detecting Moderate Demented stages because it delivers 653 precise identification results while minimally misclassifying any samples. A significant number of Very Mild Demented cases get assigned to the Mild Demented group in addition to the 111 diagnoses which the classifier labels as non-demented based on Figure 11. Distinguishing dementia at early stages from healthy individuals remains a challenge for early intervention because both cases present similar symptoms.

#### 4.3.4 Confusion matrix of ensemble model

**Figure 12:**
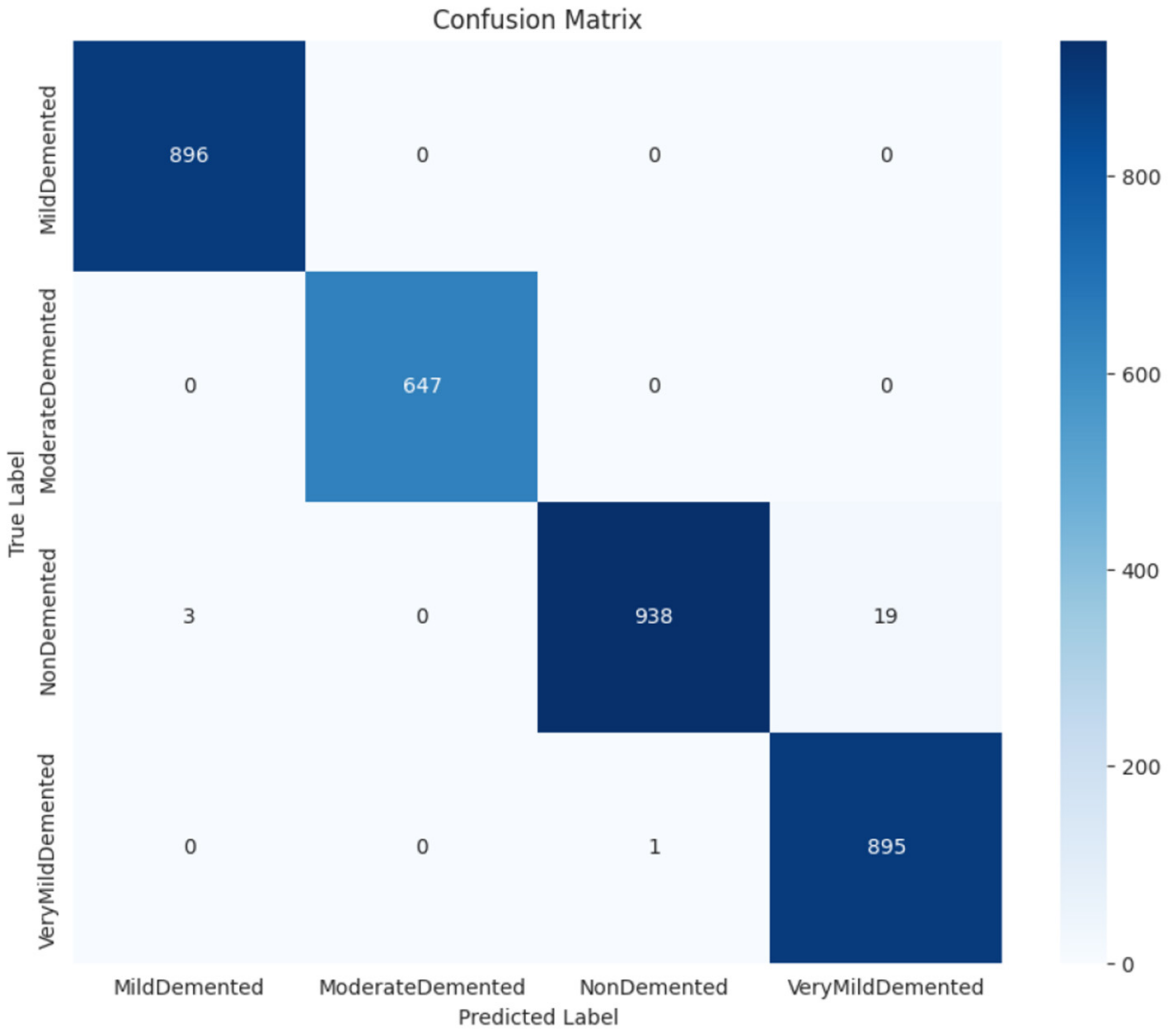
Confusion matrix of ensemble model.

Each category shows robust performance in classification based on the ensemble model where most instances fall within correct interpretations. Our analysis showed the model correctly identified 896 cases of Mild Demented and 647 cases of Moderate Demented along with 938 non-demented cases and 895 Very Mild Demented patients. The classification method shows minimal mistakes because occasional Very Mild Demented cases accidentally overlapped with non-demented cases (19 images) while other classification results were unaffected (31). The integrated ResNet50 and EfficientNet-B3 model successfully identifies different dementia stages because of its powerful feature extraction strengths. Both ResNet50 and EfficientNet-B3 contribute remarkable capabilities to classification accuracy by demonstrating strong combinations of deep learning methodology and parameter optimization capabilities. The ensemble model proves highly suitable for early-stage Alzheimer's detection through its minimal misidentification errors in identifying groups of Moderate Demented patients along with Mild Demented patients as shown in Figure 12. The ensemble model demonstrates high diagnostic accuracy which makes it suitable for automated Alzheimer's disease detection systems that would help doctors intervene early and make better medical choices. The ensemble model demonstrates superior performance by attaining maximum accuracy while making the fewest classification errors especially in subjects with Mild and Moderate Demented diagnosis. The EfficientNet-B3 performs exceptionally well in mild and moderate case identification although it displays challenges when trying to identify severe cases. The ResNet50 Model demonstrates successful operation however, its efficiency decreases when attempting to distinguish very mild Dementia from persons who do not have dementia.

## 5 External validation

To evaluate the generalization ability of the proposed ensemble model, an external validation was performed using a separate dataset comprising 6,400 MRI images representing four stages of Alzheimer's disease: Non-Demented, Very Mild Demented, Mild Demented, and Moderate Demented. The results confirm the robustness and accuracy of the model beyond the training data, demonstrating its potential for real-world clinical application (32).

The model achieved an overall accuracy of 97%, with consistently high precision, recall, and F1-scores across all classes. Specifically, the Non-Demented class yielded a precision of 0.96 and a recall of 0.94, resulting in an F1-score of 0.95. The Very Mild Demented class, which represents early-stage Alzheimer's detection, achieved perfect scores—precision, recall, and F1-score all at 1.00—though this result should be interpreted with caution due to the relatively small sample size (*n* = 10). The model also performed well on the Mild Demented and Moderate Demented categories, achieving F1-scores of 0.97 and 0.96, respectively as depicted in the Table 6 below.

**Table 6:** Performance metrics on external validation dataset.

**Class**	**Precision**	**Recall**	**F1-score**	**Support**
Mild Demented	0.96	0.94	0.95	145
Moderate Demented	1.00	1.00	1.00	10
Non-Demented	0.97	0.98	0.97	513
Very Mild Demented	0.96	0.96	0.96	356
Accuracy			0.97	1,024
Macro Avg	0.97	0.97	0.97	1,024
Weighted Avg	0.97	0.97	0.97	1,024

Macro and weighted averages for all metrics were uniformly 0.97, indicating that the model maintains consistent performance across both balanced and imbalanced class distributions. These results suggest that the ensemble model, which combines ResNet-50 and EfficientNet-B3, is capable of accurately distinguishing between Alzheimer's disease stages even when evaluated on data not seen during training.

The results are promising, but the limited number of samples in some classes—especially Very Mild Demented—warrants further validation using larger, clinically diverse datasets. Future work will focus on subject-level validation using datasets with patient identifiers, clinical metadata, and imaging protocols to assess the model's robustness in practical diagnostic environments.

## 6 Comparison with state-of-the-art

This research demonstrates how recent developments improve disease detection models and dataset capabilities and classification metrics when compared to current field-leading detection approaches. Research using deep learning algorithms ResNet50, EfficientNet, VGG16, and DenseNet has evaluated Alzheimer's disease classification from MRI scans with different degrees of achievement. The application of CAM-CNN on MRI scans with VGG19 and ResNet101 network models produced a 98.85% accuracy outcome where ResNet101 provided better performance than VGG19. The combination of EfficientNet-B2 with VGG16 allowed researchers to produce a model that reached 97.35% accuracy through transfer learning applications. Individual use of ResNet50 in previous research reached an accuracy of 80.14% yet displayed spaces where its classification accuracy might be enhanced. Research results using multiple models including VGG16 and DenseNet121 with ResNet50 demonstrated an accuracy level of 84.67 percent which indicates the requirement for better ensemble strategies. The research introduces an ensemble model that joins ResNet50 with EfficientNet-B3 to improve classification outcomes in a major way. The proposed model delivers 99% overall performance accuracy because Mild Demented, Moderate Demented, and Non-Demented classes achieve precision, recall and F1-score values of 1.00. Feature extraction capabilities of EfficientNet-B3 reveal its superiority over ResNet50 since individual assessments show precision at 0.95 vs. 0.87 and an F1-score of 0.99. To surpass benchmarked models this research generated an ensemble method that brings together beneficial characteristics from EfficientNet-B3 and ResNet50 including their optimized architecture and deep feature learning ability. Its high classification accuracy makes this approach a promising option for automated Alzheimer's detection while enabling better medical decision support particularly during early diagnosis. A summary of these two methods appears in Table 7.

**Table 7:** Comparison on the basis of aspects.

**Ref No**	**Year**	**Technique used**	**Number of classes**	**Name of classes**	**Accuracy**
(4)	2024	VGG19 and RESNET 101 with CAM-CNN	4	• Non-Dementia • Without Dementia • Very Mild Dementia • Mild Dementia • Moderate Dementia	98.85%
(7)	2023	Ensemble of EfficientNet-B2 and VGG-16	4	• Mild Demented • Moderate Demented • Non-Demented • Very Mild Demented	97.35%
(9)	2024	Using various architectures like VGG 16, VGG 19, Dense Net 121	5	• Binswanger Dementia • Hemorrhagic Dementia • Multi-infarct dementia • Strategical dementia subcortical dementia	84.67%
(10)	2024	Using deep learning techniques	4	• Mild Demented • Moderate Demented • Non-Demented • Very Mild Demented	80.14%
(15)	2024	Using ResNet, Dense Net, and Efficient Net	4	• Mild Demented • Moderate Demented • Non-Demented • Very Mild Demented	75.06%
Proposed model		Ensemble Model of Resnet 50 and Efficient Net-B3	4	• Mild Demented • Moderate Demented • Non-Demented • Very Mild Demented	99.32%

Several recent studies have contributed valuable insights into the development of intelligent diagnostic systems, which support the objective of this research. For instance, Zhang et al. (33) demonstrated the clinical benefits of precision imaging techniques in neurosurgical applications, highlighting the importance of targeted image-guided interventions in neurological disorders, a concept that aligns with the need for accurate neuroimaging analysis in Alzheimer's disease. Yin et al. (34) proposed an EEG-based emotion recognition system using autoencoder feature fusion and MSC-TimesNet, which exemplifies the utility of deep learning in neurocognitive data interpretation. Similarly, Tian et al. (35) introduced a novel self-supervised learning model for binocular disparity estimation, indicating the growing potential of self-supervised frameworks that could be extended to medical imaging applications such as Alzheimer's classification. Furthermore, Xiao et al. (36) presented a large-scale machine learning-based dementia risk model tailored to elderly populations with depression, providing a strong clinical basis for integrating predictive analytics in Alzheimer's risk assessment. Zhu (37) explored memory impairment detection through computational intelligence in substance abuse patients, reinforcing the relevance of machine learning in cognitive disorder diagnostics. Zhan et al. (38) investigated brain strain analysis using *in-vivo* and simulation data, underlining the value of biomechanical modeling in neurodegenerative research. Li et al. (39) applied machine learning to diagnose sarcopenia using sEMG signals, showing the adaptability of ML in aging-related disease detection. Lastly, Xiang et al. (40) employed a systems biology approach to explore potential therapeutic mechanisms in Alzheimer's, offering complementary biological insights that support a multimodal understanding of the disease. Together, these works underscore the feasibility and importance of leveraging advanced machine learning, neuroimaging, and multimodal integration strategies—paralleling the aims of our ensemble learning-based framework using ResNet-50 and EfficientNet-B3 for Alzheimer's diagnosis and disability assessment.

## 7 Discussion

Research development centers on building an ensemble model for Alzheimer's disease detection while showcasing its value for clinical assessments. The proposed model extends clinical abilities of neurologists and radiologists through its accuracy enhancement and robustness while facilitating timely precise diagnostic procedures that minimize human error and enhance early treatment strategies. The absence of patient-level demographic data, including age and gender, limits the model's ability to analyze performance variations across different population subgroups. Future work will utilize clinically annotated datasets to enhance interpretability and fairness and use datasets that allow patient-wise splitting to ensure proper generalization. The lack of patient identifiers prevented subject-level data splitting. Consequently, the model may have been exposed to highly correlated adjacent slices across training and test sets, increasing the risk of overfitting and overestimating performance. Although augmentation and splitting were carefully performed, the absence of subject identifiers may result in correlated slices from the same subject appearing in different data subsets, potentially impacting generalization. Through implementation in hospital imaging platforms the ensemble model functions as a medical decision tool which enables specialists to detect Alzheimer's disease manifestations at different stages confidently. Due to the absence of raw volumetric MRI files and acquisition metadata, advanced corrections such as N4 bias field correction could not be applied, which may affect intensity uniformity across slices. Since the dataset was pre-augmented and lacks original raw scans, it may not be suitable for standalone testing or external benchmarking. This restricts our ability to fully assess generalization and may introduce bias if augmentation artifacts influenced the model. Deep learning methods showcase their potential to outperform conventional diagnostic methods through the successful ensemble architecture which unites ResNet50 and EfficientNet-B3 networks. A key limitation of this work is the absence of imaging acquisition metadata, such as sequence types and scanner specifications, as the dataset was sourced from a publicly available platform (Kaggle) that did not include these details. This limits our ability to assess the model's robustness across different clinical imaging conditions. The enhanced accuracy of combined model identifications results in increased abilities to distinguish dementia's early stages from standard brain abnormalities thereby enabling prompt medical care. The improved diagnosis system reliability comes from better misclassification control which decreases false-positive and false-negative outcomes leading to incorrect diagnosis. Medical imaging is undergoing significant change through artificial intelligence as studies demonstrate the practical benefits of automatic Alzheimer's disease detection on a wide scale basis. Due to the lack of publicly available documentation the possibility of synthetic or unverified image generation cannot be ruled out, and this represents a significant limitation in terms of compliance and reproducibility. To ensure broader applicability and robustness, future work will involve validating the model on external datasets Deep learning-based models demonstrate clinically appropriate applications in patient workflows for early detection and personalized treatment development which leads to better neurodegenerative disease outcomes. Further, the proposed ensemble model can serve as an assistive tool for radiologists by providing automated classification of Alzheimer's disease stages from MRI scans. This can help flag early-stage or high-risk patients for further investigation. However, it should not replace expert interpretation. The model may produce false positives or false negatives, especially in very mild or atypical cases. Therefore, recommendation in its integration with standard clinical workflows, cognitive scoring systems, and physician review to ensure accurate diagnosis and decision-making.

## 8 Conclusion

Using MRI high-resolution scans, the research team developed an ensemble deep learning diagnostic system which performed with 99% accuracy in detecting Alzheimer's disease. The model utilized ResNet-50 to extract efficient features and EfficientNet-B3 to classify robustly while remaining effective against challenges in medical imaging applications. Precise model training and evaluation became possible through the reliable annotations and diverse high-quality image dataset which contained 33,984 images. Preprocessing methods performed through normalization, rescaling, and noise removal improved the model quality for enhanced robustness. The model demonstrated superior performance as shown through precision and recall scores together with F1-score and area under the ROC curve metrics during comprehensive evaluations across all stages of Alzheimer's disease. Our model achieved consistent training and validation accuracy improvements which converged at 99.32% with minimal overfitting observed in loss plots thus, proving its strong generalization potential. Analysis of the confusion matrix demonstrated that the model produced accurate results for both Mild and Moderate cases along with non-demented cases and achieved commendable accuracy when identifying Very Mild Demented cases. The research data shows that the ensemble model delivers strong diagnostic capabilities for Alzheimer's detection across severe disease manifestations. High-quality data alongside deep learning produces better diagnostic accuracy according to the research findings. Its performance quality makes the model suitable for clinical use because it provides essential medical decisions to doctors for early disease detection and ongoing care regulation. Further studies must evaluate both model optimization and implementation across multiple clinical settings as part of broader application validation.

## Data Availability

The original contributions presented in the study are included in the article/supplementary material, further inquiries can be directed to the corresponding authors.
